# A new large ‘silesaur’ specimen from the ?Late Triassic of Zambia; taxonomic, ecological and evolutionary implications

**DOI:** 10.1098/rsos.250762

**Published:** 2025-07-16

**Authors:** Jack Lovegrove, Kimberley E.J. Chapelle, Brandon R. Peecook, Paul Upchurch, Paul M. Barrett

**Affiliations:** ^1^ Earth sciences, University College London, London, England, UK; ^2^ Fossil Reptiles, Amphibians and Birds Section, Natural History Museum, London, UK; ^3^ Department of Anatomical Science, Stony Brook University, Stony Brook, NY, USA; ^4^ Evolutionary Studies Institute, University of the Witwatersrand Johannesburg, Johannesburg, Gauteng, South Africa; ^5^ Division of Paleontology, American Museum of Natural History, New York, NY, USA; ^6^ Idaho Museum of Natural History & Department of Biological Sciences, Idaho State University, Pocatello, ID, USA

**Keywords:** Triassic, archosaur, silesaur, Ntawere, evolution

## Abstract

‘Silesaurs’ are bird-line archosaurs that either represent the sister clade to dinosaurs or a paraphyletic grade of early ornithischians. Here, we describe a partial silesaur femur, NHMUK PV R37051, from the Ladinian/Carnian Ntawere Formation of Zambia. This femur is notable for being one of the largest silesaur specimens yet known, both globally and from the Ntawere Formation. The description of this new specimen means that two out of the three largest silesaur individuals are now known from the latter stratigraphic unit. A detailed morphological and osteohistological description of the specimen is presented alongside comparisons with other silesaur femora. Although NHMUK PV R37051 cannot be confidently referred to the only named Ntawere silesaur, *Lutungutali sitwensis*, it is also not morphologically distinct enough to justify erecting a new taxon. Furthermore, osteohistological data rule out a simple ontogenetic explanation for these large Ntawere silesaurs. This taxonomic uncertainty suggests that a more conservative approach is needed when scoring *L. sitwensis* into phylogenetic datasets. The existence of these large silesaurs challenges our understanding of dinosaur size evolution and its implications for the very early stages of their rise to ecological dominance.

## 1. Introduction

The phylogenetic affinities of silesaurids have been heavily debated. They were originally considered as members of a clade (Silesauridae) that formed the immediate outgroup to Dinosauria [1–3], but more recent analyses have recovered them as a paraphyletic grade of early ornithischian dinosaurs [4–6]; for a detailed review of this debate, see [7]. Although the relationships and monophyly of this group remain contentious, a core group of taxa is consistently recovered as part of this clade or grade, and we use the informal term ‘silesaurs’ to refer to these taxa, hereafter.

Historically, early diverging non-dinosaurian dinosauromorphs, including silesaurs, have usually been considered as small-sized members of the Middle–Late Triassic faunas in which they occur [8]. Most of these taxa have femoral lengths of ≤ 15 cm, with the largest well-known taxon, *Silesaurus opolensis*, achieving a maximum femoral length of 21 cm [8,9]. However, there have been recent challenges to this paradigm. In 2015, Barrett *et al.* published on an unnamed, partial silesaur femur NHMUK PV R16303 from the Lifua Member of the Manda beds from southwestern Tanzania. Historically, these beds were considered to be Anisian (Early–Middle Triassic) in age, based on now-defunct biostratigraphy, but are now considered to be near the Ladinian/Carnian boundary (see [10,11]). With an estimated femoral length of 345 mm, NHMUK PV R16303 would have been approximately 1.6 times longer than the largest known *S. opolensis* specimen, showing that at least some early diverging dinosauromorphs reached substantially larger sizes [12]. NHMUK PV R16303 is likely a contemporary of the much smaller silesaur *Asilisaurus kongwe* ([3,13] others), and it is possible that this larger individual represents a later ontogenetic stage of this taxon [12]. In 2017, Peecook *et al.* [10] reported on an even larger fragmentary femur (NHCC LB54) from the Ntawere Formation of Zambia (?Ladinian–Carnian), with an estimated complete length of 370 mm, or approximately 1.8 times that of the largest *S. opolensis* femur. It was not possible to refer this specimen to *Lutungutali sitwesis*, the only named silesaur taxon from this formation, due to the lack of overlapping elements with the holotype (a nearly complete pelvic girdle and caudal vertebrae; see [10,14]). This is unfortunate, as the Ntawere Formation has yielded an assemblage of approximately 25 isolated silesaur femora with a wide size range (reconstructed lengths of 60−366 mm), which might represent either a relatively complete ontogenetic sequence of a single taxon or an unusual diversity of different-sized ‘silesaurs’ within this ecosystem [10].

This article describes a second large silesaur femur, NHMUK PV R37051, from the Ntawere Formation of Zambia, which has remained undescribed since its discovery in the early 1960s, and discusses its implications for our understanding of avemetatarsalian diversity and ecology within the upper Ntawere fauna.

### 1.1. Institutional abbreviations

NHMUK, Natural History Museum, London, United Kingdom; NHCC, National Heritage Conservation Commission, Lusaka, Zambia

### 1.2. History of discovery

NHMUK PV R37051 was collected during a joint Natural History Museum/University of London expedition to Zambia (then still Northern Rhodesia) and Tanzania that took place between June and October 1963 [15]. According to the unpublished field notes of Alan J. Charig, it was found approximately 3.5 miles north of Sitwe in the upper Luangwa valley, at a site identified as ‘field locality 15E of Drysdall & Kitching [16]’. Locality 15E refers to an area dubbed ‘Archosaur Vlei’ in Charig’s fieldnotes, which was named for the abundance of small archosaur remains at this site. Many of the localities bearing the field designation ‘15’ are ‘vleis’, an Afrikaans term for a small seasonal lakebed, and represent small areas where loose, isolated elements weathered out of the mudstones during the wet season, enabling surface collection [15,16]. Unfortunately, the exact location of ‘Archosaur Vlei’ was not recorded by Charig: as a result, it is not possible to determine whether it corresponds to any of the sites visited by more recent collecting efforts in the area [10]. It is also important to note that Drysdall & Kitching [16] used their ‘locality number 15’ to refer to a large area (approximately 7.6 km^2^) of upper Ntawere Formation outcrop in the vicinity of Sitwe, meaning that the specimens recorded from this locality might not have been collected in close proximity to one another. For example, the holotype of the stahleckeriid dicynodont *Zambiasaurus submersus* comes from locality 15A, the ‘Anomodont Brae’, which is described as a large section of steep hillside exposure that is clearly distinct from the shallow low-lying vlei of locality 15E [17]. This geographic ambiguity also makes it impossible to determine whether these specimens are from the same stratigraphic level. NHMUK PV R37051 was not given a unique field number but was included among various other bone fragments recorded in Charig’s notes as ‘batch 15E/6’. This specimen was not identified and prepared until 2015 and 2017, respectively. However, its date of discovery means that it would have been one of the first silesaur specimens ever recovered, although its significance was not realized until much later.

## 2. Material and methods

### 2.1. Phylogenetic analysis

NHMUK PV R37051 was added to the data matrix of Müller [18], which was chosen as it samples a wider array of silesaur taxa than other early avemetatarsalian matrices. In the original matrix, *L. sitwensis* was scored for various maxillary, femoral and tarsal characters following the hypodigm for this material proposed by Martz & Small [19], in addition to the pelvic characters that can be scored directly from the holotype. As these referrals were made without justification, apart from all the specimens being from locality 15 (but see above), given the lack of anatomical overlap or association between these specimens, scorings based on referred specimens were removed, restricting *L. sitwensis* to the holotype. Scorings for the other taxa in this matrix were left unaltered. The final dataset, included in the electronic supplementary material, contains 282 characters and 74 operational taxonomic units (OTUs).

All phylogenetic analyses were conducted in TNT v. 1.6 [20]. Following the analytical protocols set out by Müller [18], characters 4, 13, 18, 25, 63, 82, 84, 87, 89, 109, 142, 166, 174, 175, 184, 186, 190, 201, 203, 205, 209, 212, 225, 235, 236, 239, 250 and 256 were treated as additive/ordered. All characters were equally weighted, and *Euparkeria* was set as the outgroup. The most parsimonious trees (MPTs) were found using the New Technology search algorithm, with ratchet and drift enabled, followed by a round of traditional TBR search. Both an equally weighted (EWP) and two extended implied weighting (EIW) analyses (*K* = 12 and *K* = 6, see [21] for justification) were run in which the new technology search MPTs were used as the starting trees. One unstable OTU was pruned from the MPTs *a posteriori* (*Sacisaurus*), and a strict consensus tree was generated. A symmetric resampling analysis was conducted to test node support across the tree. This analysis was run using a traditional search, a *p*-value of 0.33 and 1000 replicates. Two trees were generated by this analysis, one in which nodes with a GC (groups present/contradicted) value <10 were set to collapse and a second in which this threshold was reduced to 1.

### 2.2. Femoral length estimation

The reconstructed total length of the femur was estimated using a linear regression equation. This was calculated using R (v. 4.4.2) and plotted using the ggplot2 [22] and ggpmisc [23] packages. An expanded version of the Barrett *et al*. [12] dataset was used for this analysis, containing measurement data from 59 avemetatarsalian femora. Measurements for the 28 additional femora in the expanded dataset were sourced from the literature (see electronic supplementary material for individual references). As the distal end of NHMUK PV R37051 is absent, femoral length was calculated as a function of its proximal long-axis length alone (table 1). In addition to estimating the total length of NHMUK PV R37051, total lengths for a selection of fragmentary femora were also re-estimated using measurement data from the literature and the same regression equation. These femora were selected based on previously published estimates in the literature, themselves based on the Barrett *et al*. [12] dataset and methodology, ensuring consistency in calculation.

**Table 1.** NHMUK PVR37051 measurements of the specimen before histological sectioning.

maximum proximal mediolateral width (MPW)	48 mm
dorsoventral length of preserved portion (L)	77 mm
preserved maximum distal width (MDW)	36 mm
maximum distal height (without trochanter) (MDH)	19 mm
maximum distal height (with trochanter) (MDHT)	26 mm
maximum proximal height (MPH)	21 mm

### 2.3. Osteohistological analysis

NHMUK PV R37051 was sectioned near the proximal metaphysis at the level of the fourth trochanter using standard methodology for producing osteohistological sections from palaeontological specimens [24]. Nomenclature for the various bone tissues and structures present follows that of de Buffrénil *et al*. [25]. High-magnification and composite images were taken using a Nikon Eclipse LV100POL microscope under plain and cross-polarized light with a one-fourth plate lambda filter. Composite images were processed using Nikon NIS-Elements AR (v. 5.20.02) and Nikon NIS-Elements BR (v. 5.24.03) imaging software. The Extended Depth Focus function was used to autofocus and z-stack photomicrographs to improve the image quality.

## 3. Results

### 3.1. Morphological description and comparisons

**Figure 1.**
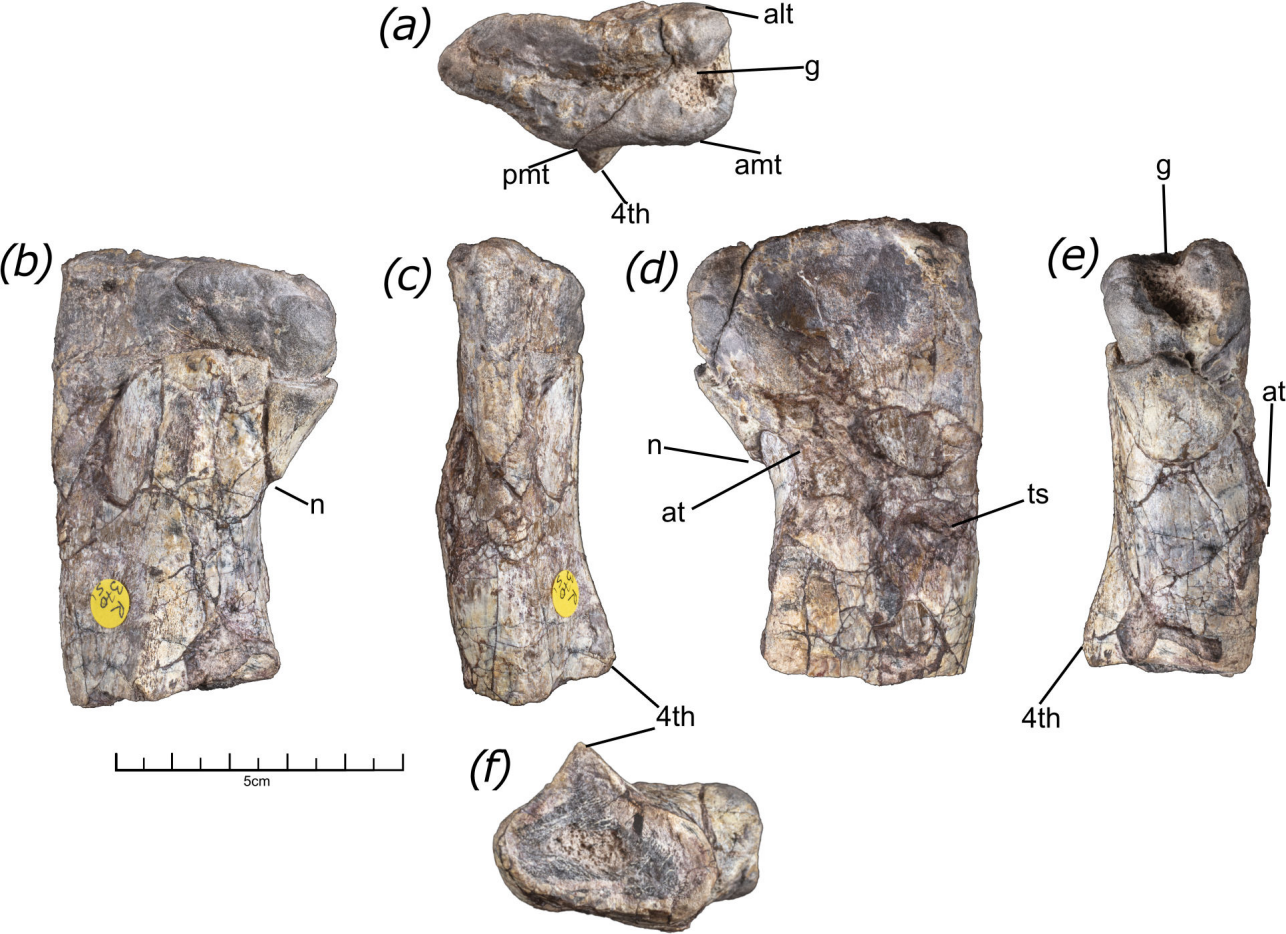
Morphology of NHMUK PV R37051, the proximal end of a silesaur femur in: (*a*) proximal view, where anterior is towards the top of the page, (*b*) posterior view, (*c*) lateral view, (*d*) anterior view, (*e*) medial view and (*f*) distal view, with posterior towards the top of the page. Features: at, anterior trochanter; alt, anterolateral trochanter; amt, anteromedial trochanter; g, proximal groove; n, notch; pmt, posteromedial tuber; ts, trochanteric shelf; 4^th^, fourth trochanter.

NHMUK PV R37051 comprises the proximal portion of a left femur, which is broken at approximately the midpoint of the fourth trochanter. It has suffered minor taphonomic damage, including some cracking and displacement of the bone cortical surface. In the proximal view, the femur has a sub-triangular outline, tapering to an acute point posterolaterally (figure 1*a*). A deep, prominent groove extends approximately mediolaterally across the proximal surface. The presence of this groove is considered a synapomorphy of Silesauridae in some analyses [3,26], although a poorly developed proximal groove is present in some other archosaur taxa, e.g. *Nundasuchus songeaensis* [27]. This groove follows the overall shape of the proximal surface and turns slightly anteriorly in its lateral portion, with a distinct inflection point that is level with the posteromedial tuber. This contrasts with the condition in *A. kongwe, Silesaurus opelensis* and NHCC LB45 in which the groove extends in an approximately straight line across the proximal surface of the femur [10,13,28].

In NHMUK PV R37051, there is no clear separation between the dorsolateral trochanter and femoral head. The facies articularis antitrochanterica (FAA) is absent, as also occurs in *S. opolensis* [28], but in contrast to *Lewisuchus admixtus, Amansaurus nesbitti* and *A. kongwe* where this feature is present, although it is strongly reduced in the latter two taxa [13,29,30]. Three distinct tuberosities are present on the proximal end: a low, but distinct, posteromedial tuber positioned on the posteromedial surface of the femur; a rounded anteromedial tuber at the anteromedial corner and a less well-defined anterolateral tuber. The anteromedial tuber appears to be the largest of the three tubera, with the posteromedial and anterolateral tubers being subequal in size. A shallow concavity is present between the posteromedial and anteromedial tubers. The surface between the anterolateral and anteromedial tubers is damaged, with the proximal groove extending into a deep concavity that is sub-rectangular in anteromedial view. While the proximal groove may have been partially visible in anteromedial view, like the condition present in *A. kongwe* [13], the damage to this surface makes it hard to know for certain. As most of the articular surface of the femur head has been damaged, it is impossible to determine if this surface was straight or convex.

In posteromedial view, the dorsal margin of the greater trochanter is straight and sharply defined, forming a 90° angle with its lateral margin. This contrasts with the condition in the unnamed Manda silesaur NHMUK PV R16303, in which the dorsal margin is rounded and descends distally to form the sloped surface of the FAA. In NHMUK PV R37051, ventral to the femoral head, a slight notch or step is present due to the femoral head being medially offset relative to the femoral shaft. Although this feature might have been accentuated by damage, this notch is another potential synapomorphy of Silesauridae [3,26]. The damage to the articular surface of the femoral head creates the appearance of a ledge proximal to this notch. In NHMUK PV R16303, a well-defined ridge extends ventrally from the posteromedial tuber (‘medial ridge’ of [12]). In NHMUK PV R37051, a low swelling appears to be present in the position of this ridge; however, the bone surface in this area is broken, which may be obscuring the development of this feature. Further distally, the fourth trochanter is present as a sharp, prominent ridge that arises from the centre of the shaft’s posteromedial surface. The fourth trochanter of this specimen appears to extend further proximally than in the other silesaur femora described from the Ntawere Formation [10].

In the anterolateral view, the proximal part of the anterolateral surface is gently concave, with the medial rim of this concavity formed by the anterolateral tuber, which creates a low ridge. A faint anterolateral scar is present, similar to that in *A. kongwe* [13]. The anterolateral tuber is very weakly developed relative to that in the unnamed Manda silesaur NHMUK PV R16303, in which it is a large swelling [12]. In NHMUK PV R37051, the anterior trochanter is positioned distal to the anterolateral tuber and forms a low ridge confluent with the femoral shaft along its whole length, similar to the condition seen in *A. kongwe* [13]. The cleft separating the proximal tip of the anterior trochanter that is seen in some other silesaurs, such as *Amanasaurus nesbitti* and *Eucoelophysis baldwini* [30–32], is absent. The distal part of the anterior trochanter merges into the trochanteric shelf, a ridge that extends mediolaterally across the anterolateral surface of the femur, although the central portion of this shelf has been damaged. The presence of this shelf differs notably from the condition in other Ntawere Formation silesaur femora in which it is absent [10]. Previous studies have found the fusion of the anterior trochanter and trochanteric shelf to be a strong indicator of skeletal maturity [33].

The femur is broken at a point midway along the fourth trochanter, revealing the latter’s distinctly triangular cross section. The preserved portion of the fourth trochanter shows that it was raised and crest-like. This is similar to the condition observed in other silesaurs, except for *Gondwanax paraisensis*, where the fourth trochanter is greatly reduced [18]. Excluding the fourth trochanter, the femoral shaft has an elliptical cross section, which is compressed anteroposteriorly. The short portion of the preserved femoral shaft curves medially relative to the proximal part of the femur. This suggests that, when complete, the femur was likely sigmoidal in anterior or posterior view, similar to the condition seen in other Zambian silesaur specimens, as well as *S. opolensis* and *A. kongwe* [10,13,28].

### 3.2. Osteohistological description

**Figure 2.**
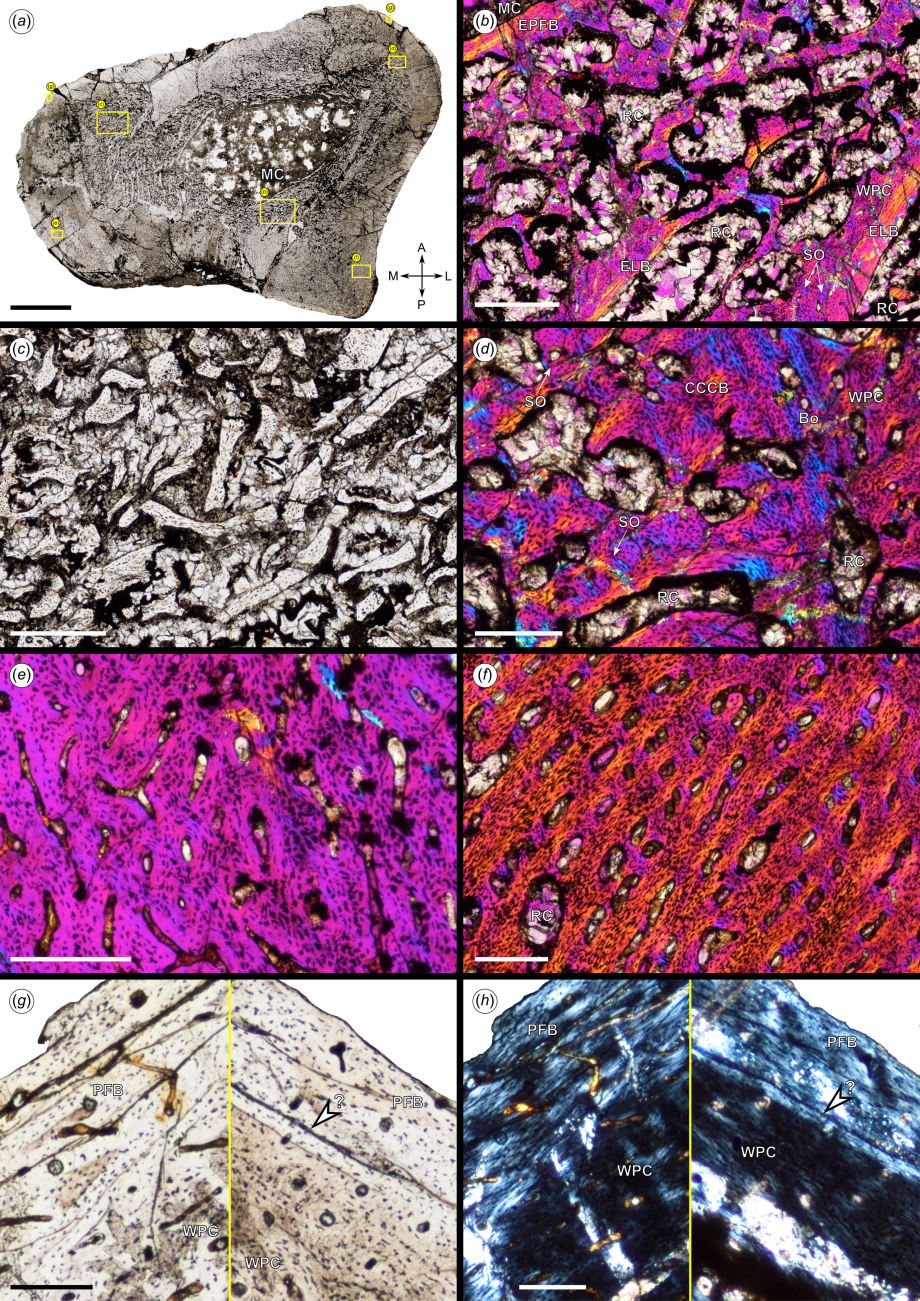
Osteohistology of NHMUK PV R37051, partial left femur. (*a*) The overall transverse section under normal light showing the open medullary cavity and breakage. Yellow boxes indicate the position of close-ups illustrated in (*b*)–(*h*). (*b*) Close-up of perimedullary region and inner cortex under cross-polarized light showing endosteal bone, coarse cancellous bone, woven-parallel complex bone tissue and scattered secondary osteons. (*c*) Close-up of middle cortex under normal light showing broken trabeculae in coarse cancellous bone. (*d*) Close-up of middle cortex under cross-polarized light showing compact, coarse cancellous bone separated from the outer WPC cortex by a boundary consisting of a band of avascular, lacunae-rich bone. (*e*) Close-up of middle cortex under cross-polarized light showing woven parallel complex and vascularization. (*f*) Close-up of middle cortex in the fourth trochanter under cross-polarized light showing woven-parallel complex with large patches of woven-fibered bone and longitudinal vascular canals arranged in rows. (*g*) Close-up of an outer cortex and subperiosteal surface under normal light showing the possible line of arrested growth (white arrow), decrease in vascularization and increase in parallel-fibered bone tissue. (*h*) Close-up of an outer cortex and subperiosteal surface under polarized light showing the possible line of arrested growth (white arrow) and change in fibre orientation from woven-parallel complex to the parallel-fibred bone. Abbreviations: A, anterior; Bo, boundary; CCCB, compact coarse cancellous bone; ELB, endosteal lamellar bone; EPFB, endosteal parallel-fibred bone; L, lateral; M, medial; MC, medullary cavity; P, posterior; PFB, parallel-fibred bone; RC, resorption cavity; SO, secondary osteon; WPC, woven-parallel complex. Scale bars: a, 5000 μm; b and c, 500 μm; d and e, 250 μm; g, m and h, 125 μm.

The medullary cavity is open, and its margin is lined with a thin layer of endosteal bone (see figure 2*a* and the top right corner of 2*b*). The latter is avascular and represented by features consistent with parallel-fibred bone (PFB: defined as no lamellae visible in the matrix of the tissue; collagen fibres parallel to the outer contours of the bone and the presence of spindle-like or flat osteocyte lacunae oriented parallel to the general direction of the collagen fibres). The inner cortex comprises coarse cancellous bone with numerous medium- to large-sized resorption cavities throughout. Some of the larger resorption cavities are lined with endosteal bone (figure 2*b*), represented by PFB or lamellar bone (LB) tissue (defined as visible lamellae in the matrix with spindle-like or flat osteocyte lacunae oriented parallel to the general direction of the collagen fibres). The trabeculae between the resorption cavities consist of a mixture of PFB tissue and woven-fibred bone (WFB) tissue (defined as fibres in matrix with no preferential orientation, with randomly oriented large osteocyte lacunae), with a few scattered longitudinal primary osteons (figure 2*b*), forming a woven-parallel complex (WPC: a mix of woven-fibred and lamellar components in the form of primary osteons). A few secondary osteons can also be seen scattered throughout the inner cortex.

This coarse cancellous bone structure extends into the middle cortex in the anteromedial and anterolateral parts of the section, where broken trabeculae can be seen (figure 2*c*). The anterior part of the section is broken and displaced, making it difficult to confirm the osteohistology in this part of the cortex. Small resorption cavities extend into the middle and outer cortex in most of the lateral half of the section. The anterolateral part of the middle cortex comprises compact coarse cancellous bone (CCCB: a remodelled, centripetal deposit of endosteal parallel-fibred or lamellar tissues in inter-trabecular spaces leading to a ‘disorganized’, complex histological structure). A few secondary osteons can be seen in the CCCB (figure 2*d*), and a clear boundary separates the CCCB from the outer cortical bone. This boundary consists mainly of a difference in tissue types, except for one region where it is formed by a band of avascular, globular lacunae-rich bone (figure 2*d*). As the section was taken from the metaphysis, this could explain the presence of CCCB because the latter is a transitional tissue. In the medial to posterolateral parts of the section, the middle cortex consists of WPC with large patches of WFB (figure 2*e*). The tissue is highly vascularized, with longitudinal primary osteons and some anastomoses. In the lateral half of the fourth trochanter, the number of osteocyte lacunae increases and WFB is more prevalent. The longitudinal canals are arranged in rows that are oriented anterolaterally to posteromedially. The tissue fibres also change in orientation. This is probably due to the mechanical stresses placed on the fourth trochanter.

The outer cortex is similar in microstructure to the middle cortex, except for the sub-periosteal surface. Most of the outer margin of this surface has been eroded away, making it difficult to determine the microstructure; however, in some areas, a slight change can be seen. There appears to be a decrease in vascularization towards the sub-periosteal surface, along with a reduction in the number of osteocyte lacunae, which appear to become more flattened and organized, forming more patches of LB and PFB and fewer WFB (figure 2*g*). Viewing the section under polarized light shows a change in fibre orientation in some parts of the sub-periosteal surface, indicating a transition in tissue type (figure 2*h*).

No growth marks (annuli or lines of arrested growth) are visible in the cortex. One possible growth mark can be seen in the anterolateral part of the section, near the sub-periosteal surface. However, the osteohistological section is badly cracked and exhibits some diagenetic alteration, making it difficult to confirm this.

Based on the high degree of vascularization and WFB in the WPC throughout the cortex, along with the lack of remodelling or visible growth marks and the slight tissue transition near the sub-periosteal surface, it is suggested that this individual was a late-juvenile or early sub-adult at the time of death (which was either just under or just over one year in age).

### 3.3. Phylogenetic results

**Figure 3.**
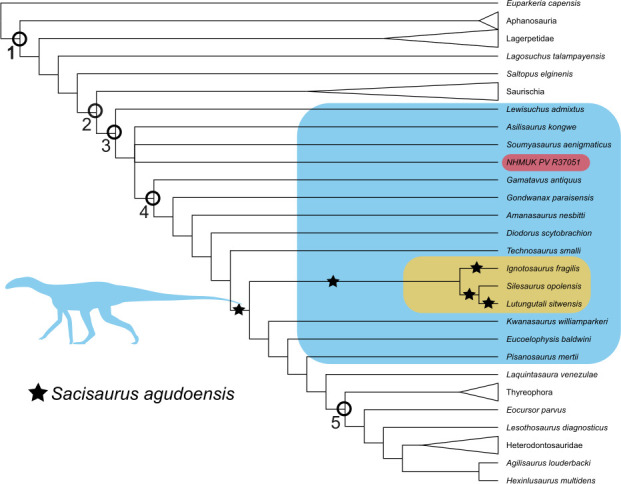
The phylogenetic position of NHMUK PV R37051 (highlighted in pink) within the Avemetatarsalia, a strict consensus tree generated from the extended implied weighting analysis (*K* = 12). ‘Silesaur grade’ taxa are highlighted in blue, and the clade Silesauridae is highlighted in gold. The recovered positions of the taxon pruned from the tree, *Sacisaurus agudoensis*, are marked with black stars. Highlighted clades are (1) Avemetatarsalia, (2) Dinosauria, (3) Ornithischia, (4) Sulcimentisauria and (5) Saphornithischia. Silhouette sourced from phylopic *Asilisaurus* (modified) by Scott Hartman (CC BY 4.0).

**Figure 4.**
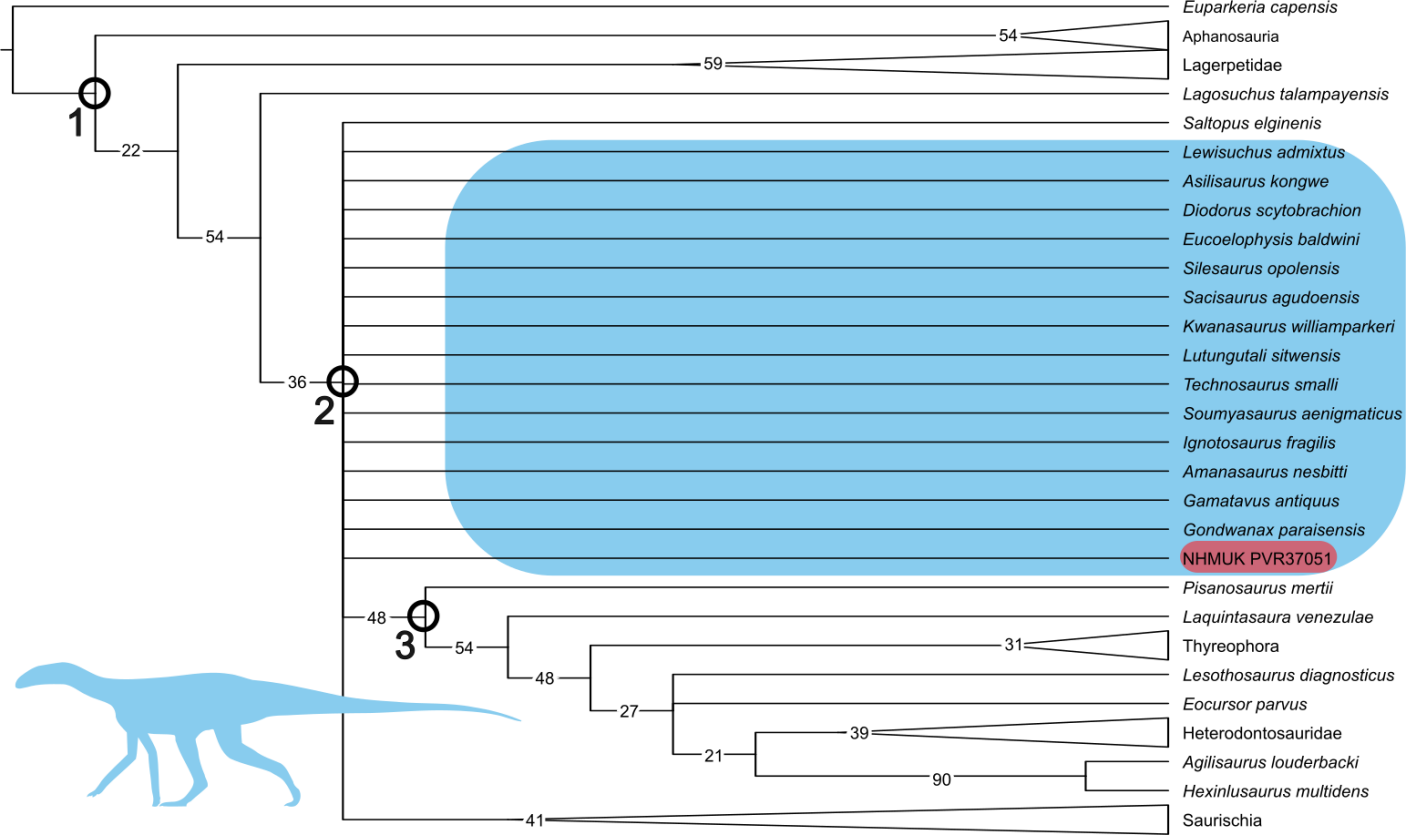
The phylogenetic position of NHMUK PV R37051 (highlighted in pink) within the Avemetatarsalia collapsed strict consensus tree generated from the extended implied weighting analysis (*K* = 12). Numbers in black are GC values calculated from a symmetric resampling analysis; clades where this value was less than or equal to 10 have been collapsed. ‘Silesaur grade’ taxa are highlighted in blue, although note that *Pisanosaurus mertii* is not included as it is recovered in its traditional position as a basal ornithischian rather than as part of this grade. Highlighted clades are (1) Avemetatarsalia, (2) Dracohors and (3) Ornithischia. Several clades, including Dinosauria and Sulcimentisauria, have collapsed into the polytomy at the base of Dracohors. Silhouette sourced from phylopic *Asilisaurus* (modified) by Scott Hartman (CC BY 4.0).

The EWP phylogenetic analysis recovered 3024 MPTs with lengths of 1067 steps. The EIW analysis with *K* = 12 recovered 135 MPTs with lengths of 43.568, and the analysis with *K* = 6 recovered 324 MPTs with lengths of 68.802. The position of *Lutunugutali sitwensis* is better supported by the EIW (*K* = 12) analysis, so it was used as the basis for the main figures (figures 3 and 4) and discussion in this article; see the electronic supplementary material for other trees. The strict consensus tree (figure 3) is similar in overall topology to those obtained from previous analyses of this dataset [5,6,18,30], with the most noticeable similarity being that the ‘traditional’ members of Silesauridae are recovered not as a clade but as a paraphyletic grade of basal ornithischians; see the electronic supplementary material. This result is unsurprising considering the small number of modifications we have made to the existing dataset. However, if the following stem-based definition of Silesauridae is used—‘all archosaurs closer to *Silesaurus opolensis* than to *Heterodontosaurus tucki* and *Marasuchus lilloensis*’ [34]—Silesauridae would be retained as the clade containing *S. opolensis, Ignotosaurus fragilis*, *L. sitwensis* and possibly *Sacisaurus agudoensis*.

NHMUK PV R37051 is recovered in a polytomy with *A. kongwe*, *Soumyasaurus aenigmaticus* and Sulcimentisauria. This suggests that it is one of the earliest diverging silesaurs and a close relative of *A. kongwe,* consistent with the geographic proximity of the two taxa. *Lutungutali sitwensis* was recovered as a member of Silesauridae and a sister taxon to the clade’s namesake *S. opolensis* (see figure 3).

A symmetric resampling EWP analysis (*p* = 0.33, replicates = 1000) was run to investigate the levels of support for individual nodes within the tree. The GC values indicate that support for the key hypothesis gleaned from variations of this dataset (that silesaurs are ornithischians) is weak. This is best visualized by setting the strict consensus tree to collapse nodes with a GC of <10 (see figure 4). Even at this low GC threshold, the internal relationships within Dracohors are no longer resolved, although the clade itself is comparatively well supported (GC = 42). Dracohors was defined by Cau [35] as ‘the most inclusive clade containing *Megalosaurus bucklandii* but excluding *Marasuchus lilloensis*’, although the latter taxon has since been considered a junior synonym of *Lagosuchus talampyensis* [36]. A large polytomy at the base of Dracohors contains the silesaurs, *Saltopus elginensis*, Ornithischia and Saurischia. Interestingly, *Pisanosaurus mertii* becomes the earliest diverging ornithischian (GC = 48) rather than collapsing into a polytomy with silesaurs.

### 3.4. Femoral length estimations

The linear regression recovered a strong and statistically significant correlation between proximal long axis (*w*), usually equivalent to the mediolateral width of the proximal femur and total femur length (*l*):

.$$l=4.52+5.44w(P<0.001,\;\;\;R^{2}=0.80)$$

The *R*^2^ value of 0.80 suggests that the proximal long axis length explains a significant proportion of the variation in total femoral length, although (unsurprisingly) not as much as the combination of proximal and distal long axis lengths used by previous studies (*R*^2^ of 0.91) [12]. The new, expanded dataset estimates slightly lower overall femoral lengths than previous analyses (table 2), with the new values being 2%−9% shorter than those estimated by previous studies (37) [10,30]. Based on the 95% confidence intervals, the new dataset gives more precise estimates of femur length than previous studies (although it is impossible to say if it is more accurate, as the actual length of these femora will never be known). The trio of large unnamed East African silesaurs (NHMUK PV R37051, NHMUK PV R16303 and NHCC LB54) are substantially larger than all other known silesaurs, even with the slightly lower length estimates obtained by this study. The holotype of *S. opolensis* (ZPAL AbIII/361) is usually reconstructed with a body length of approximately 2.1 m (see, e.g. the skeletal diagram in Piechowski & Dzik [38]). Assuming NHMUK PV R37051 scaled isometrically and had similar proportions, it would have approached 3 m in length, and NHCC LB54 would have reached a length of approximately 3.5 m. As with many questions about the biology of these animals, more complete fossil remains are required to test these hypotheses.

**Table 2.** Specimens with lengths estimated using linear regression from figure 5.

specimen	predicted femur length (in mm)	previous estimated femur length (in mm)
NHMUK PV R16303	314 ± 24	346 ± 50 [12]
NHCC LB54	336 ± 27	370 [10]
NHMUK PV R37051	266 ± 18	NA
PVSJ 898 (holotype of *Dromomeron gigas*)	173 ± 9	190 (37)
CAPPA/UFSM 0374 (holotype of *Amanasaurus nesbitti*)	119 ± 8	121.5 [30]

**Figure 5.**
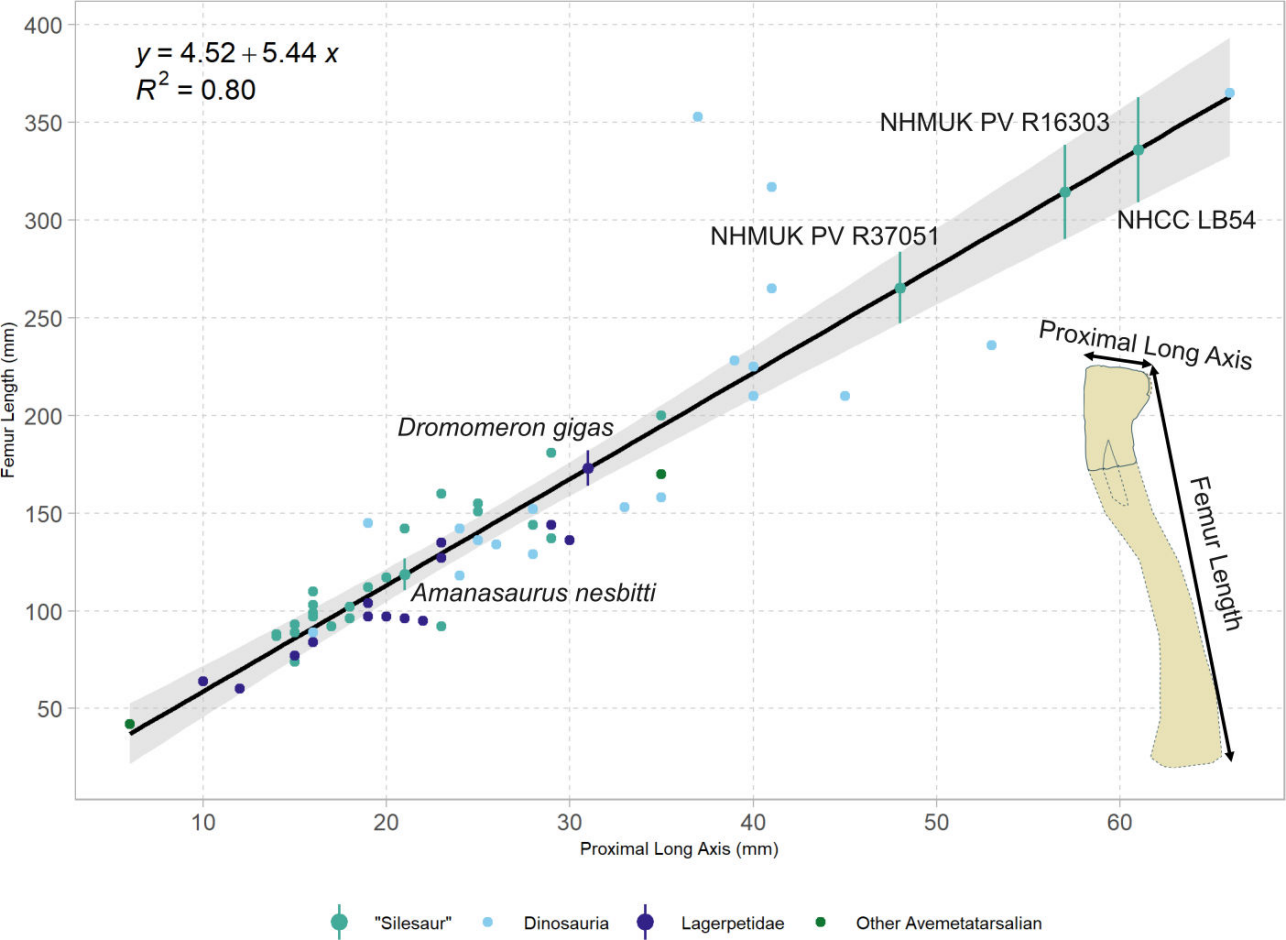
Femoral length regression for early diverging avemetatarsalians. The linear regression equation (top left) was calculated based on the dataset plotted on the graph (dots coloured by phylogenetic group). The linear regression line is plotted in black with a 0.95 confidence interval displayed in grey. Raw data are available in the electronic supplementary material.

## 4. Discussion

### 4.1. Taxonomic implications

Several minor anatomical features differentiate NHMUK PV R37051 and the previously described Ntawere Formation silesaur femora, namely the presence of a trochanteric shelf and the greater proximal extents of both the fourth and anterior trochanters in the former [10]. Unfortunately, the presence of a trochanteric shelf appears to be ontogenetically variable within silesaurs, given that this shelf and anterior trochanter are both absent in the smallest known *A. kongwe* femur (NMT RB169), while the second smallest specimen has an anterior trochanter but lacks a trochanteric shelf (NMT RB220), suggesting that these features developed in sequence during ontogeny [33]. In *S. opolensis,* the presence of a trochanteric shelf is variable in medium-sized individuals, although one is present in the largest femora and absent in the smallest [9]. This pattern has been interpreted as evidence of sexual dimorphism, with females attaining higher average body sizes and developing accessory ossifications due to an increase in calcitonin levels during puberty [9]. However, as discussed in Griffin & Nesbitt [33], it is more probable that this represents individual variation in the onset of ontogenetic changes, with the apparent bimodal distribution being an artefact of the small sample size [9]. Due to ontogenetic variation and sequence polymorphism in the development of the trochanteric shelf in *A. kongwe* and *S. opolensis*, it is unwise to assign taxonomic significance to the presence of a trochanteric shelf in NHMUK PV R37051, even though its similarity in size to NHCC LB54 suggests that these individuals were at similar ontogenetic stages. The fragmentary nature of most known Ntawere silesaur femora makes it difficult to quantify the variation in the positions of the anterior and fourth trochanters. The features distinguishing NHMUK PV R37051 from the other Ntawere Formation silesaur femora could be due to intraspecific or ontogenetic variation.

The upper Ntawere Formation and the middle/upper assemblage of the Lifua Member of the Manda Beds were deposited at approximately the same time in basins less than 200 km apart [10]. As might be expected based on their geographical proximity, the tetrapod faunas found in these formations are very similar, with four genera being common to both formations (the traversodontid cynodont *Luangwa*, the trirachodontid cynodont *Cricodon*, the stahleckeriid dicynodont *Sangusaurus* and the capitosaur ‘*Stanocephalosaurus’*; [10]). Because of this, there is a possibility that NHMUK PV R37051 might be referable to *A. kongwe*, the unnamed Lifua Member silesaurid [13] or to the same taxon as NHMUK PV R16303, the ‘giant’ unnamed silesaur from the same unit [12]. A combination of proximal femoral characters is considered diagnostic for *A. kongwe*, following Nesbitt *et al*. [39]. These are the presence of an FAA; equally developed anterolateral, anteromedial and posteromedial tubera; a proximally pointed anterior trochanter and the possession of a trochanteric shelf in large individuals. The presence and expansion of the proximal tuberosities in NHMUK PV R37051 are comparable to those in *A. kongwe,* although its anterolateral tuber differs from the swollen condition present in NHMUK PV R16303 [12]. The development of the anterior trochanter and trochanteric shelf in NHMUK PV R37051 is comparable to that of *A. kongwe*. However, NHMUK PV R37051 differs from *A. kongwe* and NHMUK PV R16303 in one major detail, namely in lacking an FAA. The presence of this feature is considered taxonomically informative for silesaurs and has been included in the diagnoses of *A. kongwe* and *A. nesbitti* [13,30]. Ontogenetic variation in the development of the FAA has not been documented in *A. kongwe* [33]. Its absence in NHMUK PVR37051 is, therefore, strong evidence that this specimen is not referrable to *A. kongwe*.

### 4.2. Phylogenetic implications

While scoring NHMUK PV R37051 into the Müller [18] dataset, its scores were compared to those of other silesaurs. It is noteworthy that Müller followed Martz & Small [19] in scoring *L. sitwensis* for several femoral, maxillary and tarsal characters, although the holotype only consists of a pelvis and caudal vertebrae [14]. These additional scores appear to be based on the creation of a hypodigm containing the holotype plus other silesaur material described by Peecook *et al*. [10]. However, this hypodigm assumes that only one silesaur taxon is present in the Ntawere Formation. Although most Middle–Late Triassic formations that preserve silesaur remains currently contain a single named taxon, for example *Diodorus syctobracion* from the Timezgadiouine Formation [40] or *A. kongwe* from the Lifua Member [13], this likely reflects the rarity of their fossil remains and the recency of the clade’s description and definition, rather than a genuine ecological signal. Indeed, formations that have been more intensively sampled/studied in recent years have been found to contain several coeval silesaur taxa, for example *Gamatavus antiquus* and *G. paraisensis*, from the lower Santa Maria Formation [18,41]. The possibility that there are multiple silesaur taxa in the Ntawere Formation fauna, therefore, cannot be ruled out (see above).

Our phylogenetic analysis recovers *L. sitwensis* as unusually deeply nested among silesaurs, given its stratigraphic age, and as only distantly related to the earlier diverging NHMUK PV R37051. This challenges the assumption that all Ntawere formation silesaur specimens are referable to the same taxon and highlights the value of taking a more conservative approach when scoring taxa into phylogenetic datasets. However, this result must be caveated by the extremely fragmentary nature of NHMUK PV R37051, and indeed, most silesaur taxa. The discovery of more complete specimens that overlap anatomically with the holotype of *L. sitwensis* is necessary to confirm the presence of a second silesaur taxon in the Ntawere Formation.

The results of the symmetric resampling analysis suggest that the Müller [18] dataset only weakly supports the ‘ornithischian silesaur’ hypothesis. It is worth noting that most of the analyses that have supported this hypothesis have used a variation of this same dataset [5,6,18,30] or incorporated it as a part of a supermatrix approach [4]. These results suggest that the problems identified with other datasets focused on avemetatarsalian relationships are also present in this one [7,42,43], indicating that avemetatarsalian (and, in particular, early dinosaurian) relationships remain in a state of flux [7].

### 4.3. Osteohistological comparisons

Osteohistological data are available for several other silesaurs, including *S. opolensis* [44], *A. kongwe* [33] and three unnamed silesaurid specimens from northeastern Zambia [10]. In *S. opolensis*, the sectioned elements (femur, tibia, metatarsal, ribs) show a fast growth rate, cortical bone composed of WPC (PFB in ontogenetically older specimens), little secondary remodelling, few or no growth marks (except in one of the larger tibiae) and dense longitudinal vascularization that decreases towards the subperiosteal surface (indicating maturation). All these characteristics are consistent with the features present in NHMUK PV R37051. When comparing the femora, those sampled for *Silesaurus* are all smaller than NHMUK PV R37051 (diameters of approx. 23 mm or less, as opposed to approx. 35.7 mm in NHMUK PV R37051). In these *Silesaurus* femora, the medullary cavity is large and open, and the perimedullary region lacks coarse cancellous bone in sections taken from the mid-diaphyseal region, but has cancellous tissue in sections taken from the metaphyseal region. The latter condition is more similar to that in NHMUK PV R37051, which was sectioned in the metaphyseal region, but it should be noted that the cancellous bone in *Silesaurus* is highly remodelled, with thin cortical walls formed by a poorly vascularized PFB deposit with scarce primary osteons. All three sectioned *Silesaurus* femora display a reversal marking the cortex/medullary transition, along with an avascular annular transition zone. The cortical thickness seen in *Silesaurus* femora is thinner than that in NHMUK PV R37051 (maximum of 18%−19.5% versus 58%, respectively). None of *the Silesaurus* specimens exhibit growth marks.

The sectioned elements of *A. kongwe* (femur, humerus, tibia, fibula) are not from the largest available specimens, although the largest femora are still only approximately 70% the size of NHMUK PV R37051 [25,33]. Overall, the osteohistology of *Asilisaurus* consists of WFB, longitudinal primary osteons with extensive anastomoses in some areas and numerous osteocyte lacunae with a slight decrease in density towards the outer cortex. The tibia shows CCCB in the inner cortex, separated from outer cortical bone by a boundary with similar micromorphology to that observed in the femur of NHMUK PV R37051. The cortical thickness in *Asilisaurus* is described as comparable to that of *Silesaurus* and, therefore, thinner than in NHMUK PV R37051. None of the sectioned elements of *Asilisaurus* shows growth marks.

NHMUK PV R37051 has a predicted femoral length of 266 ± 18 mm. Consequently, it is one of the largest recorded silesaur specimens, with NHCC LB54 from the Triassic Ntawere Formation of northeastern Zambia being the largest (with an estimated femoral length of 336 ± 27 mm). Two smaller silesaurid femora are also known from this unit, NHCC LB78 and LB79 (with estimated lengths of 160 mm). NHCC LB78 and LB79 share similar osteohistological features. Their cortical thickness is thinner (approx. 36%) than that of NHMUK PV R37051. The dominant tissue type is mainly PFB with longitudinal vascular canals, few anastomoses and vascularization density decreases as it extends towards the sub-periosteal surface. A prominent reversal line is visible in both NHCC LB78 and LB79. The largest femur, NHCC LB54, has a cortical thickness of approximately 46%, is dominated by PFB with an increase in WPC (i.e. FLB) towards the inner cortex, is heavily vascularized with abundant reticular anastomoses throughout the cortex and exhibits sparse secondary remodelling in the inner cortex (less than in NHCC LB78 and LB79). No growth marks are visible in any of these femora.

Early branching pterosauromorphs, such as *Dromomeron* [25,45], have similar femoral osteohistology to that of silesaurids and other early diverging bird-line archosaurs. This includes WFB with longitudinal and radial primary osteons in the inner cortex, with a shift to PFB with fewer osteons in the outer cortex. Growth marks were found near the subperiosteal surface of the smaller *Dromomeron* femur sampled (GR 1036). In *Dromomeron*, the tibia possesses CCCB as a result of cortical drift, with a clear boundary separating the CCCB from the outer primary cortical bone. This boundary consists of a simple difference in tissue types, except for one region where it is formed by a band of avascular, lacunae-rich bone. This boundary is similar to that observed between CCCB and the outer primary cortical bone in NHMUK PV R37051.

Finally, the dinosaur *Nyasasaurus parringtoni* has comparable gross osteohistological morphology to that of other early branching dinosauromorphs [25,46]. A humeral section from *Nyasasaurus* shows unremodelled WFB, primary longitudinal osteons with numerous anastomoses and decreases in osteocyte lacunae and vascularization density towards the outer cortex. No growth marks are visible.

In summary, all early branching dinosauromorphs possess remarkably similar gross osteohistological morphology, although all sampled silesaurid femora have thinner cortices than NHMUK PV R37051: this and other differences could be because the section of the latter specimen was taken from the metaphysis. While growth marks are rarely seen in early branching dinosauromorphs, several specimens show a similar change in tissue type towards the sub-periosteal surface, indicating a shift from a rapid to a slower growth rate, possibly indicating maturation.

Based on our osteohistological analysis, NHMUK PV R37051 appears to have been a late-stage juvenile or subadult, transitioning from a period of fast growth, characterized by high levels of vascularization and WFB, to a period of slower growth, as characterized by PFB and LB around the subperiosteal surface. A single possible growth mark is present near the periosteal surface, suggesting this individual was approximately one year old at the time of death. The other large Ntawere Formation silesaur specimen, NHCC LB54, also exhibits a prolonged period of fast growth [10], and both these specimens lack an external fundamental system, suggesting they were still undergoing somatic growth. This period of fast growth likely explains the larger size of these specimens relative to other silesaurids. Initially, it was theorized that these larger silesaur specimens might represent adult individuals of coeval silesaur taxa [12]; however, a growing body of osteohistological data for the Ntawere Formation silesaurs seems to disprove this hypothesis [10]. The histology of the smaller Ntawere Formation femora (NHCC LB78 and LB79) shows they were much slower growing than either NHMUK PV R37051 or NHCC LB54 [10]. This is the opposite pattern to that which would be predicted if these femora formed an ontogenetic sequence, as the fastest growth rate occurs early in ontogeny. Based on osteohistological characteristics alone, it would be impossible for NHCC LB78/79 to mature into either NHCC LB54 or NHMUK PV R37051 [10].

Peecook *et al*. [10] proposed three hypotheses to account for why the different Ntawere Formation silesaur femora do not seem to form an ontogenetic sequence: (1) they are different species, (2) they form a single species with extreme intraspecific variation in size and growth trajectory, or (3) they represent different populations separated in time. As described above, there are minor morphological differences between some specimens; however, these are not taxonomically significant enough to provide strong support for hypothesis 1. Unfortunately, the fragmentary nature of the currently available specimens means that identifying diagnostic traits is difficult, although the osteohistological differences between the largest and smallest femora might imply that more than one silesaur species was present. As detailed locality and stratigraphic information are absent for many of the Ntawere Formation femora, it is impossible to determine which specimens are coeval, precluding tests of hypothesis 2 or 3. However, hypothesis 2, regarding extreme individual variation or sexual dimorphism, cannot be rejected. The finding of more complete, large silesaur specimens in the Ntawere Formation, ideally including elements that overlap with those in the holotype of *L. sitwensis* (and with detailed locality information), will likely be the only way to confidently distinguish these competing hypotheses.

### 4.4. Ecological implications

**Figure 6.**
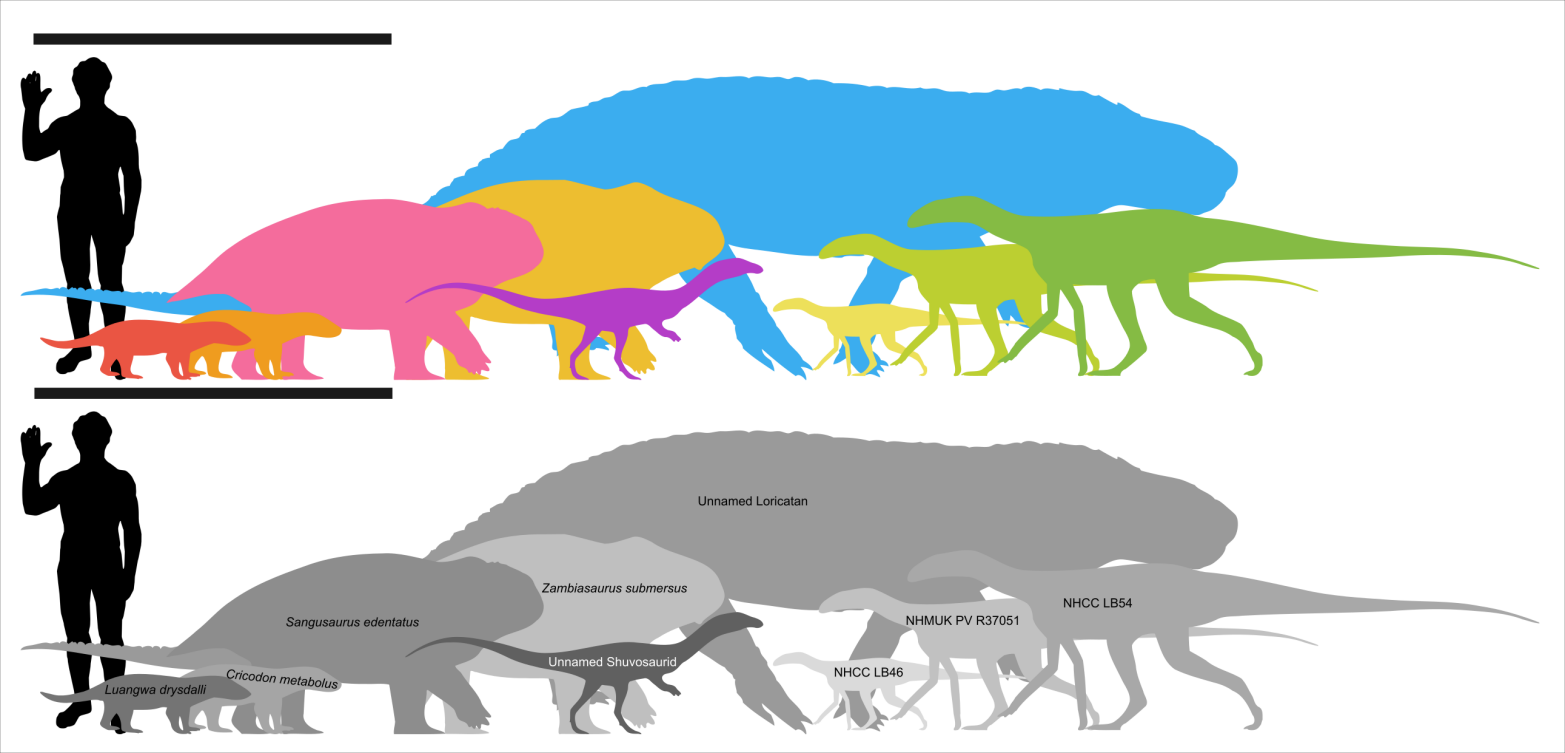
Upper Ntawere terrestrial fauna (scale bar = 2 m, human silhouette = 1.8 m). Facing left: NHCC LB46 chosen as comparable in size to the *Lutungutali* holotype. Silesaurs were scaled based on femoral length estimates from Peecook *et al*. [10] (NHCC LB46) and those calculated in this article (NHMUK PV R37051, NHCC LB54) and the proportions of the *Silesaurus opolensis* holotype. Synapsids were scaled based on published skull lengths, loricatans scaled based on length estimate from Peecook *et al*. [10], and shuvosaurids were scaled based on femoral shaft width comparison to *Shuvosaurus* specimens [39]. Silhouettes were modified from versions available on phylopic (left to right) *Prestosuchus* by Dmitry Bogdanov (vectorized by T. Michael Keesey (CC BY 3.0), *Massetognathus* (modified) by Micheal Tripoli (CC BY 4.0), *Placerias* (modified) by Tasman Dixon, *Shuvosaurus* (modified) by Sarah Werning (CC BY 4.0) and *Asilisaurus* (modified) by Scott Hartman (CC BY 4.0).

Although sauropodomorphs and herrerasaurians rapidly eclipsed silesaurs in size (for example the early Norian sauropodomorph *Macrocollum* had femoral lengths of up to 365 mm [47,48]), it is important to consider silesaurs in the context of the upper Ntawere terrestrial fauna. Silesaurs have been considered minor components of largely synapsid- and pseudosuchian-dominated faunas [49]. When scaled alongside the other vertebrates from this fauna (see figure 6), it becomes clear that the larger silesaur individuals (NHMUK PV R37051 and NHCC LB54) would have been among the largest animals in this ecosystem, although the heavily built dicynodonts likely exceeded them in terms of body mass. Although some stahleckeriid dicynodonts and shuvosaurid pseudosuchians attained giant sizes, 4+ and 8+ m in length, respectively [26,50], the published Ntawere Formation material from these clades, despite being fragmentary, suggests that their representatives in this fauna were substantially smaller than their relatives from elsewhere [10,17]. Additionally, silesaurs appear to have been the most abundant archosauromorphs in the Ntawere Formation, being known from eight localities (or seven if ‘locality 15E’ overlaps with one of the more recently collected localities), one of which is a bonebed that preserves at least seven individuals [10]. Based on the abundance of their remains and the size they attained, silesaurs should be considered a significant component of the Ntawere fauna, challenging the traditional view of these East African Triassic faunas as synapsid dominated [17,51,52].

The ecological niche filled by these large silesaurs is difficult to infer based on their phylogenetic position alone. The early diverging silesaur *L. admixtus* has strongly recurved and serrated teeth indicative of carnivory, whereas later diverging taxa like *Kwanasaurus williamparkeri* have phyllodont teeth adapted for herbivory [19,53]. However, NHMUK PV R37051 is recovered as more closely related to those silesaurs with simple, conical teeth, e.g. *A. kongwe* and *S. aenigmaticus* [3,54], whose dietary preferences are not well understood. The group’s namesake, *S. opolensis*, possesses a similar dentition and was initially considered to be a herbivore based on its tooth morphology and edentulous jaw tips [28]. Subsequently, it has been regarded as a herbivore, specifically a folivore or an omnivore feeding on soft-bodied prey based on tooth microwear [55] and, most recently, a specialized insectivore with occasional piscivory, based on braincase morphology and the contents of referred coprolites [56]. This uncertainty around the dietary ecology of the most completely known, well-studied silesaur taxon emphasizes the difficulty of reconstructing the role(s) large silesaurs played in Gondwanan ecosystems.

### 4.5. Broader implications

The following discussion of the broader macroevolutionary implications is necessarily speculative due to the uncertain phylogenetic position of silesaurs. The description of this partial femur reinforces the observation that at least some silesaurs reached much larger sizes than expected based on more completely known specimens, as already shown by the descriptions of NHCC LB54 and NHMUK PV R16303. This suggests that they were more ecologically significant than previously assumed. Palaeoecological studies of Triassic sequences in Poland have found that silesaurs occupied a small but significant niche in Carnian faunas prior to the appearance of saurischian dinosaurs and maintained this niche into the Norian despite the appearance of early saurischians [49]. This is supported by the description of *A. nesbitti*, a silesaur from the Carnian of Brazil that was roughly equivalent in size to coeval sauropodomorphs [30]. The Norian–Rhaetian niche invasion/expansion by saurischians, the beginning of the ‘rise of dinosaurs’, appears to have been preceded by a smaller Carnian (or possibly Ladinian) ‘rise of silesaurs’ in which the latter established themselves as components of global terrestrial faunas [30,49]. This model raises several macroevolutionary questions: for example, if silesaurs successfully co-existed with saurischians during the latter’s initial radiation, why did they suddenly disappear at the end of the Norian? Moreover, if silesaurs are early ornithischians, why do ornithischians disappear from the fossil record in the Rhaetian only to reappear in the earliest Jurassic with a host of novel anatomical features? Is the reduction in size between the Triassic silesaurs and early Jurassic ornithischians a product of the late Triassic extinctions selecting for smaller body size?

The acquisition of relatively large body sizes by some silesaurs could have significant macroevolutionary implications, regardless of whichever phylogenetic hypothesis for silesaur relationships proves correct, but especially if they are recovered as early ornithischians. Several macroevolutionary studies have examined body size evolution in early ornithischians [57,58], with one of these identifying two ‘exceptional nodes’ among these taxa representing rapid evolutionary shifts in body size, namely an increase at the origin of Thyreophora and a decrease at the origin of Heterodontosauridae [57]. If the ‘ornithischian silesaur’ hypothesis is correct, then the inclusion of ‘large silesaurs’ in these studies would probably have shifted the position of, and/or changed the magnitude of these exceptional nodes. Indeed, the evolution of Ornithischia across the Triassic–Jurassic boundary would show a pronounced miniaturization trend from larger, early diverging silesaurs to much smaller early Jurassic ornithischians (e.g. *Eocursor parvus*, *Scutellosaurus lawleri* and *Laquintasaura venezulae*), the opposite of the pattern found by previous studies [59]. Turner & Nesbitt [59] estimated the ancestral ornithischian femur length as approximately 112 mm, less than one-third the length of the femora in large silesaurs. If large silesaurs were among the earliest diverging dinosaurs, this, in combination with the even larger early-diverging herrerasaurids, raises the possibility that the last common ancestor of dinosaurs was larger than previously assumed, with independent decreases in body size among the main dinosaur clades. This possibility is, to some extent, independent of the debate over the relationships between those clades [7].

## 5. Conclusions

NHMUK PV R37051 helps to demonstrate that silesaurs were capable of achieving larger body sizes than indicated by the most complete specimens known currently. When complete, this femur would have measured approximately 266 mm in length making it the third largest silesaur, and possibly the third largest non-dinosaurian dinosauromorph, known. Detailed morphological and osteohistological comparisons between all the Ntawere Formation silesaur femora suggest that they cannot confidently be referred to the same taxon or to *L. sitwensis*. Because of this, a more conservative approach must be taken when scoring *L. sitwensis* into phylogenetic data matrices, likely involving restricting it to the holotype, until additional material can be referred more confidently. Based on their reconstructed body sizes and relative abundance, silesaurs were likely a significant component of the upper Ntawere Formation fauna. Unfortunately, the phylogenetic position of silesaurs remains unresolved, and symmetric resampling values suggest that the dataset commonly used to support the ornithischian-silesaur hypothesis poorly resolves relationships within Dracohors. This study demonstrates the value of studying overlooked historic specimens, no matter how fragmentary, and adding their unique data to broader evolutionary studies.

## Data Availability

Scans of NHMUK PV R37051 are available on morphosource: https://www.morphosource.org/projects/000607343?locale=en. Additional datasets supporting this publication are contained in the supplementary material [60].
